# Exosomal miRNA Profiling is a Potential Screening Route for Non-Functional Pituitary Adenoma

**DOI:** 10.3389/fcell.2021.771354

**Published:** 2022-01-18

**Authors:** Liang Lyu, Haiyan Li, Cheng Chen, Yang Yu, Li Wang, Senlin Yin, Yu Hu, Shu Jiang, Feng Ye, Peizhi Zhou

**Affiliations:** ^1^ Department of Neurosurgery, Pituitary Adenoma Multidisciplinary Center, State Key Laboratory of Biotherapy/Collaborative Innovation Center for Biotherapy, West China Hospital of Sichuan University, Chengdu, China; ^2^ Laboratory of Lung Cancer, Lung Cancer Center, West China Hospital of Sichuan University, Chengdu, China; ^3^ Department of Neurosurgery, People’s Hospital of Deyang City, Deyang, China

**Keywords:** non-functional pituitary adenoma, exosome, miRNA profile, hsa-miR-486-5p, disease screening, progression, relapse

## Abstract

Non-functional pituitary adenomas (NFPAs) are one of the most prevalent pituitary adenoma subtypes. The lack of reliable screening approach for NFPAs for the insidious clinical course usually leads to delays in medical therapy and consequently worse prognosis. Hence, we employed a sequence cohort (patient: control, 6:2) and a validation cohort (patient: control, 22:8) to develop a serum exosomal miRNA profile-based method for NFPA screening and prognosis prediction. We found that a total of 1,395 kinds of human miRNA were detected. Compared with healthy donors, 18 up-regulated and 36 down-regulated miRNAs showed significant expression alterations in NFPA patients. Target genes of differentially expressed miRNAs are mainly enriched in axonogenesis and cancer-associated terms. After validation, hsa-miR-486-5p, hsa-miR-151a-5p, hsa-miR-652-3p_R+1, and hsa-miR-1180-3p were promising biomarkers for NFPA, in which miR-486-5p was the most competent one. After a median of 33 months of prospective follow-up, exosomal hsa-miR-486-5p also was an efficient predictive biomarker for progression or relapse of NFPAs. By protein-protein interaction network construction of hsa-miR-486-5p targeted genes, the core modules revealed a high possibility that exosomal hsa-miR-486-5p regulated tumor progression by epigenetic regulation of MAPK signaling pathways. In conclusion, exosomal hsa-miR-486-5p, hsa-miR-151a-5p, hsa-miR-652-3p_R+1, and hsa-miR-1180-3p are candidate biomarkers for diagnosis and screening of NFPAs. More importantly, prospective follow-up reveals that hsa-miR-486-5p can be regarded as a significant predictor for prognosis of NFPAs.

## Introduction

Pituitary adenoma (PA) is a heterogeneous entity derived from the adenohypophysis. Based on the clinical manifestations, PAs are categorized into non-functional PAs and functional PAs (FPAs) which include growth hormone (GH)-secreting adenoma (GHPA), prolactinoma, thyrotropin (TSH)-secreting adenoma, and corticotropin (ACTH)-secreting adenoma (Tjörnstrand and Nyström, 2017). From the histopathological aspect, PAs are also divided into somatotroph, lactotroph, thyrotroph, corticotroph, gonadotroph, null-cell, and plurihormonal and double adenomas according to the adenohypophyseal cell lineage (Lopes, 2017). More importantly, different subtypes have different diagnostic and therapeutic approaches. Dopamine agonists (DAs) and somatostatin analogues (SSAs) are crucial treatment options for prolactinomas and GHPAs, respectively (Andersen, 2014; Maiter and Delgrange, 2014). However, the application of DA and SSA in NFPAs is still under evaluation (Greenman et al., 2016). Therefore, differential diagnosis of PAs is a vital task for clinicians. FPAs are characterized by hormone hypersecretion. Thus, elevated GH, TSH, prolactin, or ACTH with specific clinical symptoms are valuable clues for diagnosis of FPAs, which can be used in FPA screening. At present, the diagnosis of NFPAs is drawn only after ruling out other subtypes of PAs. Meanwhile, although most PAs are benign, a significant number of PAs possess an aggressive clinical course (Di Ieva et al., 2014). With the enlargement of tumors, PAs invade the surrounding structures leading to incomplete resection and consequent poor prognosis. Different from FPAs, owing to the absence of significant clinical clues, NFPAs tend to be invasive macroadenomas at diagnosis, which may exacerbate the therapeutic outcomes and medical costs. At the same time, regular MRI scan is the first choice during the follow-up of postoperative NFPA patients, which maybe expensive and time-consuming. Thus, the development of novel screening and diagnostic method, especially non-invasive or microinvasive technique, for NFPAs would improve the accuracy of diagnosis and subsequent treatment strategy as well as the efficiency of postoperative management of NFPA patients.

Exosome offers a possible route to the perioperative management of NFPAs. Exosomes are a pool of 40–150 nm extracellular vesicles (EVs) delivered by almost all cell types (Raposo and Stoorvogel, 2013; Kalluri, 2016). Notably, similar to cytomembranes, exosomes are composed of a lipid bilayer which contains all known molecules including protein, RNA, and DNA (Kalluri, 2016; Smalheiser, 2007). It is reported that exosomes are present in the serum, saliva, breast milk, urine, and cerebrospinal fluid (Wang et al., 2019) and involved in multiple physiological and pathological processes (Katakowski and Chopp, 2016). Besides, owing to the structure of exosomes, they are relatively stable in body fluids (Ge et al., 2014). This makes the cargos as well as the membrane proteins of exosomes desired biomarkers for screening of diseases (Lyu et al., 2019; Zhang et al., 2019). microRNA (miRNA) is a class of small noncoding RNA that have an essential role in regulation of gene expression at the post-transcriptional level (Bartel, 2009). miRNAs constitute only a small portion of human genome, but control about 30% of coding genes (Zöller, 2016). By binding mostly to the 3′ untranslated region, miRNAs repress protein expression via degradation of targeted mRNAs. Abnormal expression of miRNA has been reported in a range of tumors, including PAs (Wierinckx et al., 2017). Therefore, miRNAs, such as circulating miRNAs, have recently been linked to the diagnosis and prognosis for cancers (Mitchell et al., 2008; Németh et al., 2019). Different from circulating miRNAs, exosomal miRNAs are promising biomarkers for cancer screening for the stability and abundance of exosomes in serum.

Hence, in this study, we would like to establish a non-invasive method for NFPA screening. Due to the ease and repeatability of sample collection, serum-derived exosomes were submitted to miRNA sequencing and microarray to detected useful exosomal miRNA biomarkers for screening of NFPA. The effects of these exosomal miRNAs on prognosis prediction were verified during long-term clinical follow-up.

## Materials and Methods

### Patient Collection and Prospective Follow-Up

All serum samples involved in this study were acquired from pathological verified NFPA patients who underwent surgery at West China Hospital of Sichuan University from August 2017 to December 2018. Samples from 10 age- and gender-matched healthy donors served as control. NFPA patients (sequence cohort and validation cohort) underwent routine preoperative evaluations, which included MRI scans with contrast enhancement for tumor size and invasiveness (Knosp grade) (Micko et al., 2015) and comprehensive endocrinological assessment for possible endocrine disorders (Lv et al., 2019). After surgery, NFPAs were categorized into null cell, gonadotroph, plurihormonal, and corticotroph adenomas according to postoperative pathological studies (Lopes, 2017).

Samples were collected 1 or 2 days before surgery at 7∼8 am after overnight fasting and extracted by centrifuging at 2500 g for 10 min. After centrifugation for two times, serum samples were stored at −80°C before exosome isolation.

NFPA patients were recruited 3 months after surgery to receive MRI scans and endocrinological assessments. Gross total resection (GTR) was achieved in patients without tumor residual and was proved by postoperative MRI scans. Also, at this stage, patients with residual were recommended to Gamma knife radiosurgery. Subsequently, MRI scans and endocrinological assessments were employed every 6 months or when symptoms deteriorated during the prospective follow-up period.

Written informed consents were obtained from all individuals involved in this study. The procedures followed ethical standards of the Declaration of Helsinki 1975, as revised in 1983 and were approved by the Biomedical Research Ethics Committee of West China Hospital of Sichuan University.

### Exosome Isolation

Exosomes were isolated by a differential centrifugation method (Zöller, 2016). Briefly, serum was centrifuged at 300 g for 10 min after diluting by phosphate buffer solution (PBS). Thereafter, supernatants were centrifuged at 2000 g for 10 min, followed by a centrifugation of 10,000 g for 30 min and another centrifugation of 10,000 g for 70 min (Hitachi, Himac CP80WX) after filtering through 0.22 μm filters (Merck Millipore). The pellet containing exosomes were resuspended in 100 μl of PBS and stored at −80°C for further experiments.

### Nanoparticle Tracking Analysis

Isolated exosomes were subjected to nanoparticle tracking analysis (NTA) for measurement of concentration and size by Particle Metrix system (ZetaView, Meerbusch, Germany). Each sample was diluted with PBS to give counts in the linear range of the instrument. The motion model of particles was recorded by camera for 60 s in triplicate. Data were collected and analyzed by ZetaView software (version 8.04.02 SP2).

### Transmission Electron Microscopic Analysis

A total of 10 μl exosome suspensions were loaded on a copper grid and desiccated for 20 min. Then, 2% uranyl acetate in water was added to the exosome layer to allow another dry overnight. After that, transmission electron microscopic (TEM) imaging was performed the next day.

### Western Blotting

Exosomes were lysed by ultrasonic machine and the protein concentrations were obtained by BCA Protein Assay (Pierce, Thermo Scientific). Extracted proteins were separated in 12% polyacrylamide gel electrophoresis and analyzed by Western blot. Proteins were transferred to PVDF membranes and incubated with CD63 (1:1,000, Abcam) and TSG101 (1:1,000, Abcam) at 4°C overnight. After being incubated with secondary antibody, the proteins were visualized by chemiluminescence (Millipore) and quantified by Fusion Solo 4 chemiluminescence imaging system (VILBER, France).

### RNA Extraction and miRNA Expression Profiling by Next-Generation Sequencing

Total RNA was extracted from exosomes by RNeasy Serum/Plasma kits (Qiagen, Hilden, Germany) according to the manufacturer’s instructions. For miRNA sequence, libraries were prepared by using the TruSeq Small RNA Library Preparation kits (Illumina, San Diego, CA) according to the instructions. The libraries were validated by Agilent 2100 bioanalyzer system (Agilent Technologies, Waldbronn, Germany). miRNA sequencing was performed using Illumina Hiseq 2500 system (Illumina, San Diego, CA, United States).

### Data Analysis for miRNA Sequencing and Bioinformatics

Clean data were generated by trimming off the 3′ adapter and low-quality reads from the raw reads using ACGT101-miR software (LC Sciences, Houston, TX, United States). Subsequently, clean data with length in 18∼26 nucleotide were reserved as unique sequences and aligned to the miRBase 22.0. The unique sequences mapping to human mature miRNAs in hairpin arms were identified as known miRNAs. The unique sequences mapping to the other arm of known specific species precursor hairpin opposite to the annotated mature miRNA-containing arm were novel 5p- or 3p-derived miRNA candidates. The unmapped sequences were mapped to the human genomes, and the hairpin RNA structures containing sequences were predicated from the flank 80 nt sequences using RNAfold software (http://rna.tbi.univie.ac.at/cgi-bin/RNAWebSuite/RNAfold.cgi). These sequences that mapped to the human genomes and predicted by RNAfold software were regarded as novel miRNAs. Additionally, next-generation sequencing (NGS) could identify miRNA variations. Thus, the miRNA variations were denominated according to the following criteria: 1) length variation at both 3′ (iso3p) and 5′ (iso5p) ends were marked as L ± n or R ± n; 2) miRNAs with mismatch inside of the sequence were sequence variants (isoSNP), which were marked as N_1_ssN_2_X_1_X_2_N_3_X_1_X_2_ (N_1_, number of mismatched nucleotides; N_2_, N_3_, location of the substitution; X_1_, original nucleotide; X_2_, substituted nucleotide).

Data normalization was conducted according to a previous study (Fu et al., 2016). According to the normalized read number, the expression levels of miRNAs were categorized as low (≤10), middle (>10 but ≤mean read number), and high (>mean read number). Calculation of differential expression between healthy donors (HDs) and NFPA patients was performed by T-test or Wilcoxon rank sum test. miRNAs with *p*-value <0.05 were considered as differentially expressed in two groups. Only mature miRNAs with high or middle expression level were used in further analyses. Statistical analyses were done using R statistical programming language (Version 4.0.3). To confirm the expressions of top differentially expressed miRNAs (DE-miRNAs) in circulation, GSE131483 dataset (Németh et al., 2019) was downloaded from Gene Expression Omnibus (https://www.ncbi.nlm.nih.gov/geo/), and preoperative samples of NFPA subtype and controls were analyzed by DESeq2 package (Love et al., 2014).

Target genes of DE-miRNA were predicted by TargetScan and miRanda databases. Predicted target genes were eliminated if context score percentile ≤50 in TargetScan algorithm or max energy ≥ −10 in miRanda algorithm. Thus, target genes in both databases were used for further analysis. Target genes enrichment analyses including annotations from Gene Ontology (GO), Kyoto Encyclopedia of Genes and Genomes (KEGG) pathway, and Disease Ontology (DO) databases were conducted by clusterProfiler package between HDs and NFPA patients, where the cutoff points of *p* value and *q* value were 0.05 and 0.01, respectively (Wu et al., 2021). Gene Ratio was defined as the ratio of the number of differentially expressed genes and total gene number in specific terms.

### Validation of Differentially Expressed miRNA by Microarray

Allowing for the small sample size of our sequence cohort, another validation cohort consisted of samples from 8 HDs and 22 NFPA patients were further analyzed by miRNA microarray. Exosomal RNA was extracted as previously described. cDNA was acquired by using the miRCURY LNA RT kits (QIAGEN) according to the instructions. The expression level of miRNA was determined by MiRCURY LNA miRNA Custom PCR Panel (QIAGEN) following the parameters: 95°C for 2 min, and subsequent 45 cycles of 95°C for 10 s and 56°C for 1 min. cel-miR-39-3p was used for internal control. Data were present as ΔCT.

### Protein-Protein Interaction Network Construction

The previously identified target-genes of hsa-miR-486-5p were submitted to STRING online tool (https://www.string-db.org/) to determine physical/functional relationship among these target genes (minimal interaction score was 0.4). Protein-protein interaction (PPI) network was constructed by Cytoscape (Version 3.8.2) and highly connected nodes (hub genes) were defined by interactive Venn diagram of the top 50 nodes with high degree, high betweenness, and high closeness using cytoHubba plugin, respectively. The most significant (top 3) clustered modules were identified by MCODE plugin, and visualized by STRINGdb (Szklarczyk et al., 2019) and igraph (Csardi and Nepusz, 2006) (https://igraph.org/r/) packages. Furthermore, enrichment analyses of genes in each module also employed the STRING online tool, and false discovery rate (FDR) < 0.05 determined by BH method was considered significant.

### Statistical Analysis

Data were expressed as the mean ± standard deviation (SD) or median. Normality test was performed before further analysis for quantitative data. Statistical comparison was carried out by the Student’s *t* tests or Wilcoxon rank sum tests. Pearson or Spearman correlation analyses were performed regarding the association of DE-miRNAs and clinicopathological factors. Linear regression model was performed for the association between hsa-miR-486-5p and hsa-miR-151-5p. Receiver-operating characteristic (ROC) curves were performed to verify the discriminability of exosomal miRNAs on NFPA diagnosis, and to identify the optimal cutoff point of hsa-miR-486-5p expression on prognosis prediction. Independent predictive factor for NFPA diagnosis was verified by binary logistic regression whereas Cox hazard regression and Kaplan–Meier tests were used for Relapse/Progression-free survival. Probability of *p* < 0.05 determined from the two-sided test was considered significant. The statistical analysis was carried out by using GraphPad Prism, version 6 (GraphPad Software, La Jolla, CA, United States) and SPSS version 17.0 (SPSS Chicago, IL, United States).

## Results

### Exosomal miRNA Analysis by Next Generation Sequencing

**FIGURE 1:**
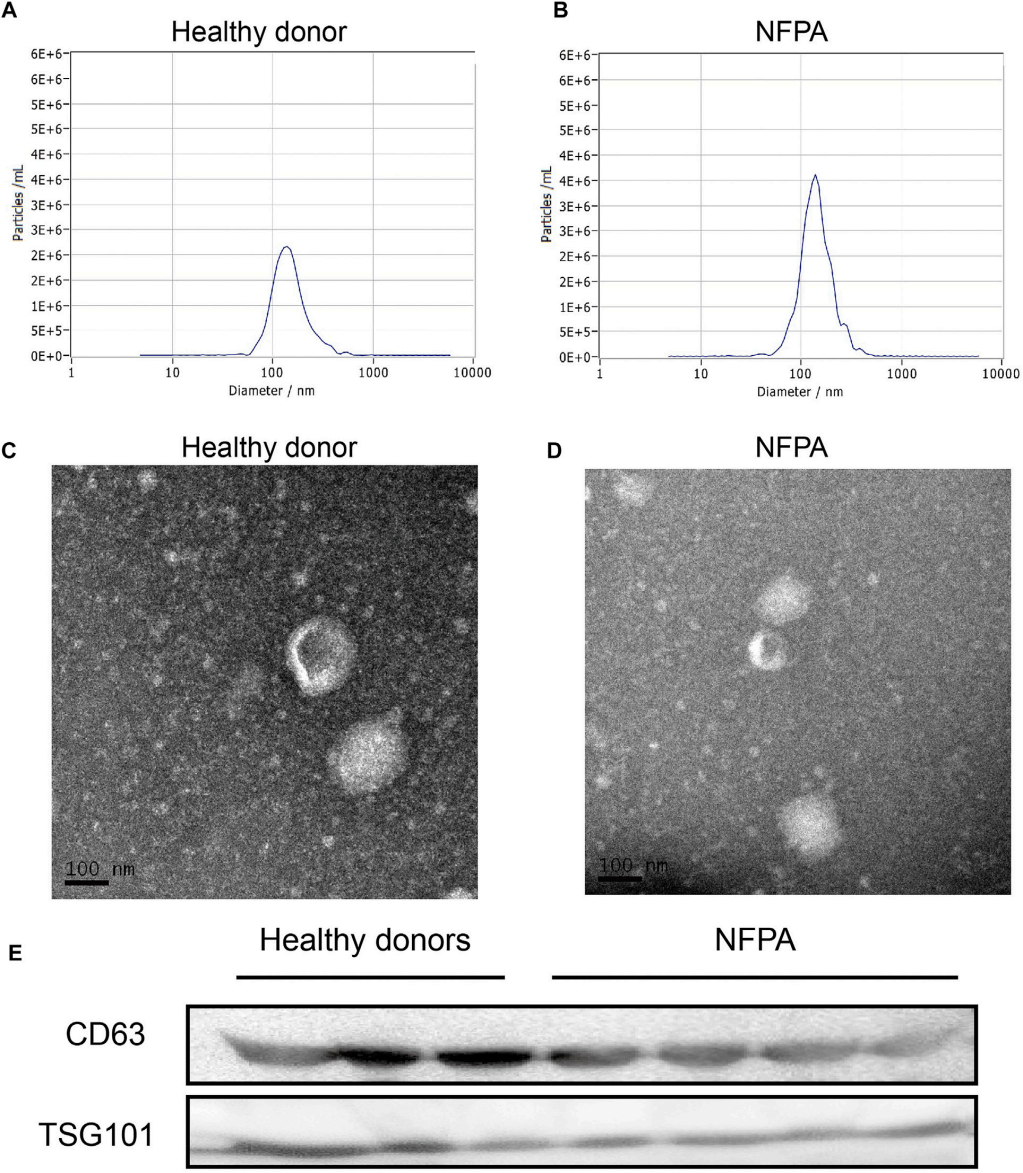
Isolation and identification of exosome. NTA data indicated that the size of isolated particles was mostly nanoscale **(A,B)** and presented a round or oval shape with central cavity on TEM **(C,D)**. They also expressed the widely accepted exosome markers (CD63 and TSG101) in Western blotting **(E)**.

First, serum exosomes were isolated from blood samples by ultracentrifugation. The median number of isolated particles was 1.1 × 10^10^ particles/ml and was consistent in HDs and NFPA patients (median, 1.1 × 10^10^ vs. 3.6 × 10^9^ particles/ml, *p* = 0.0763, Figures 1A,B). The median major peak of NTA was 136.2 nm (range, 127.4∼149.9 nm), which indicated a representative exosome size. TEM imaging detected a round or oval shape with central cavity (Figures 1C,D). Furthermore, Western blotting data revealed that these particles expressed CD63 and TSG101, which were widely accepted markers of exosome (Figure 1E). Thus, these results supported the existence of exosome in the isolated particles.

NGS was performed on 8 samples which included 3 invasive NFPA, 3 non-invasive NFPA, and 2 HDs. The total reads ranged from 3,949,997 to 17,742,877 and a total of 1395 kinds of human miRNA were detected. A total of 47.6% of detected miRNAs could map to the sequences in miRBase and genome and were regarded as known miRNAs. However, another 726 miRNAs could also map to the genome but have not been reported, which were regarded as novel miRNAs. Only 13.6% of known miRNAs were detected in high expression level, and all novel miRNAs were in middle or low expression level.

In addition, miRNA variations are categorized into three groups including sequence variants at miRNA 3′ end (iso3p), at 5′ end (iso5p), or sequence variants (isoSNP). Among known miRNAs, 204 miRNA variations were detected (39 iso3ps, 177 iso5ps, and 27 isoSNPs). There was no statistic difference about the total reads of these variations between HDs and NFPA patients (data not shown).

### Differentially Expressed miRNA in Blood Samples From Non-Functional Pituitary Adenomas

**FIGURE 2:**
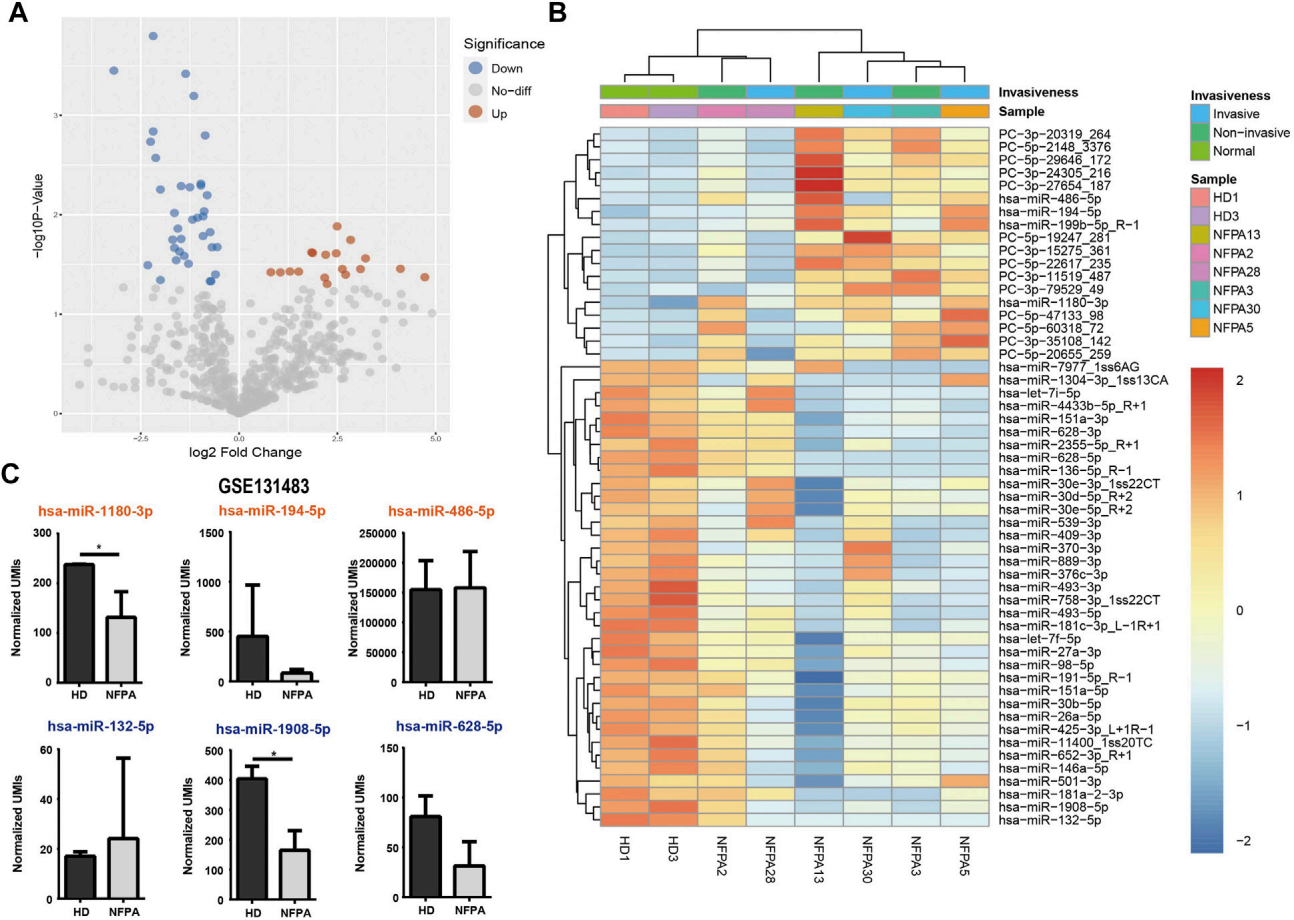
Differential expression analysis of exosomal miRNAs. Volcano plot **(A)** and heatmap **(B)** showed the 54 DE-miRNAs in NFPAs and HDs. **(C)** Circulating miRNA dataset (GSE131483) was reanalyzed for comparison of exosomal miRNA and circulating miRNA (orange: up-regulated in exosome; dark blue: down-regulated in exosome).

The differential expression analysis between NFPAs and HDs yielded a total of 54 mature miRNAs that showed significant alteration in expression, which included 18 up-regulated and 36 down-regulated miRNAs in the NFPA group (Figures 2A,B) and detailed information of DE-miRNAs is listed in Supplementary Table S1. There was no exosomal miRNA that was differentially expressed between invasive and non-invasive NFPAs (data not shown). As we know that some miRNAs may tend to be enriched in exosome, we compared the top DE-miRNAs with a circulating miRNA dataset of PAs (GSE131483). Among the three known up-regulated (hsa-miR-1180-3p, hsa-miR-194-5p, and hsa-miR-486-5p) and down-regulated (hsa-miR-132-5p, hsa-miR-1908-5p, and hsa-miR-628-5p) DE-miRNAs, only hsa-miR-1908-5p was consistently down-regulated in circulation and exosome (Figure 2C).

**FIGURE 3:**
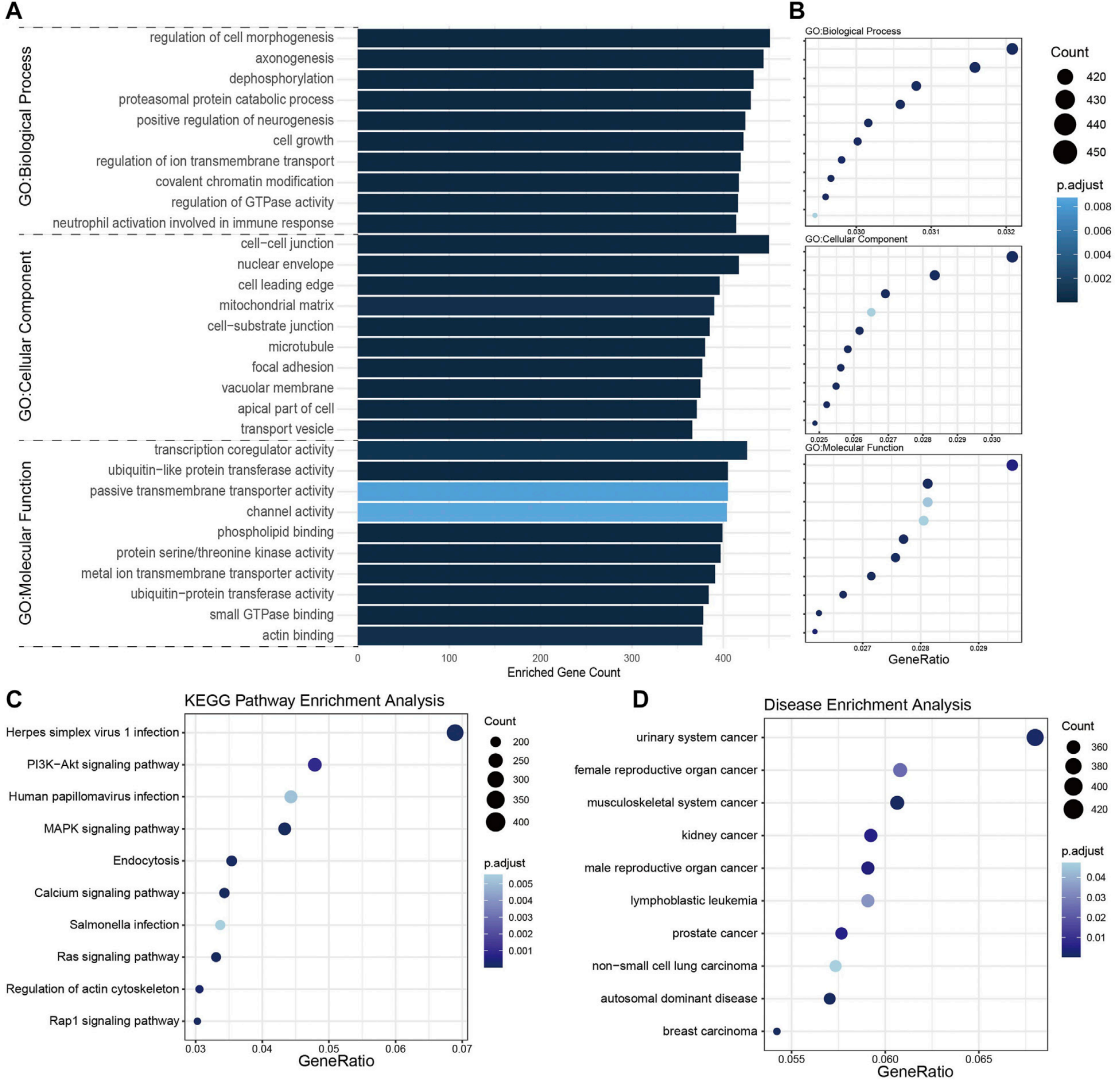
Enrichment analysis of target transcripts of DE-miRNAs. **(A,B)** GO enrichment analysis showed the top 10 terms each in biological process, cellular component, and molecular function. **(C)** KEGG pathway enrichment analysis revealed target transcripts enriched in Herpes simplex virus 1 infection and PI3K-Akt signaling pathway. **(D)** Disease ontology (DO) enrichment analysis indicated these target transcripts mainly enriched in cancer-associated terms.

To further understand the biological function of these dysregulated miRNAs, we searched the target transcripts of these DE-miRNAs in TargetScan and miRanda databases, and a total of 16,399 transcripts were identified. GO enrichment analyses revealed that target transcripts involved in regulation of morphogenesis, cell-cell junction, and cell leading edge terms, indicate that DE-miRNAs might regulate the tumor invasion and metastasis. Also, target transcripts enriched in axonogenesis and positive regulation of neurogenesis terms indicated that DE-miRNAs might affect the function of posterior pituitary in a paracrine manner, allowing for the neuroderm origin of neurohypophysis. Besides, DE-miRNAs might influence the post-translational modification of proteins that target transcripts enriched in terms like dephosphorylation, ubiquitin-like protein transferase activity (Figures 3A,B). KEGG pathway enrichment analyses revealed that DE-miRNAs might have impact on tumor growth for enrichment in PI3K-Akt and MAPK signaling pathway along with the cell growth GO term (Figure 3C). In DO terms, it was not surprising that many cancer-associated terms were highly enriched, indicating an underlying NFPA origin of these DE-miRNAs (Figure 3D). These results might serve as clues that NFPA-derived exosome may regulate tumor progression in a paracrine manner.

### miRNA Profile Differentiates Non-Functional Pituitary Adenoma Patients From Healthy Donors

Allowing for the small sample size of sequencing cohort, additional blood samples were employed for validation of 13 DE-miRNAs, which included hsa-miR-1180-3p, hsa-miR-486-5p, hsa-miR-27a-3p, hsa-let-7f-5p, hsa-miR-151-3p, hsa-miR-146a-5p, hsa-miR-652-3p_R+1, hsa-miR-194-5p, hsa-miR-30b-5p, hsa-miR-151a-5p, hsa-miR-26a-5p, hsa-miR-98-5p, and hsa-miR-199b-5p_R+1. The validation cohort consisted of samples from 8 HDs and 22 NFPA patients. The NFPA group included 13 males and 9 females with a mean age at diagnosis of 53.73 ± 9.88 years (Supplementary Table S2). The mean height and width of tumors were 2.41 ± 0.66 cm and 2.58 ± 0.86 cm, respectively, and the median anteroposterior (AP) diameter was 1.95 cm. Postoperative pathological studies revealed that null cell adenoma was the most common subtype (*n* = 9), followed by gonadotroph (*n* = 8), plurihormonal (*n* = 3), and corticotroph adenoma (*n* = 2). Invasive NFPAs accounted for 31.8% of validation cohort. However, all the validated miRNAs showed no correlation with age and gender of patients, tumor size, and morbidity of hypopituitarism in Pearson or Spearman correlation analyses (data not shown).

**FIGURE 4:**
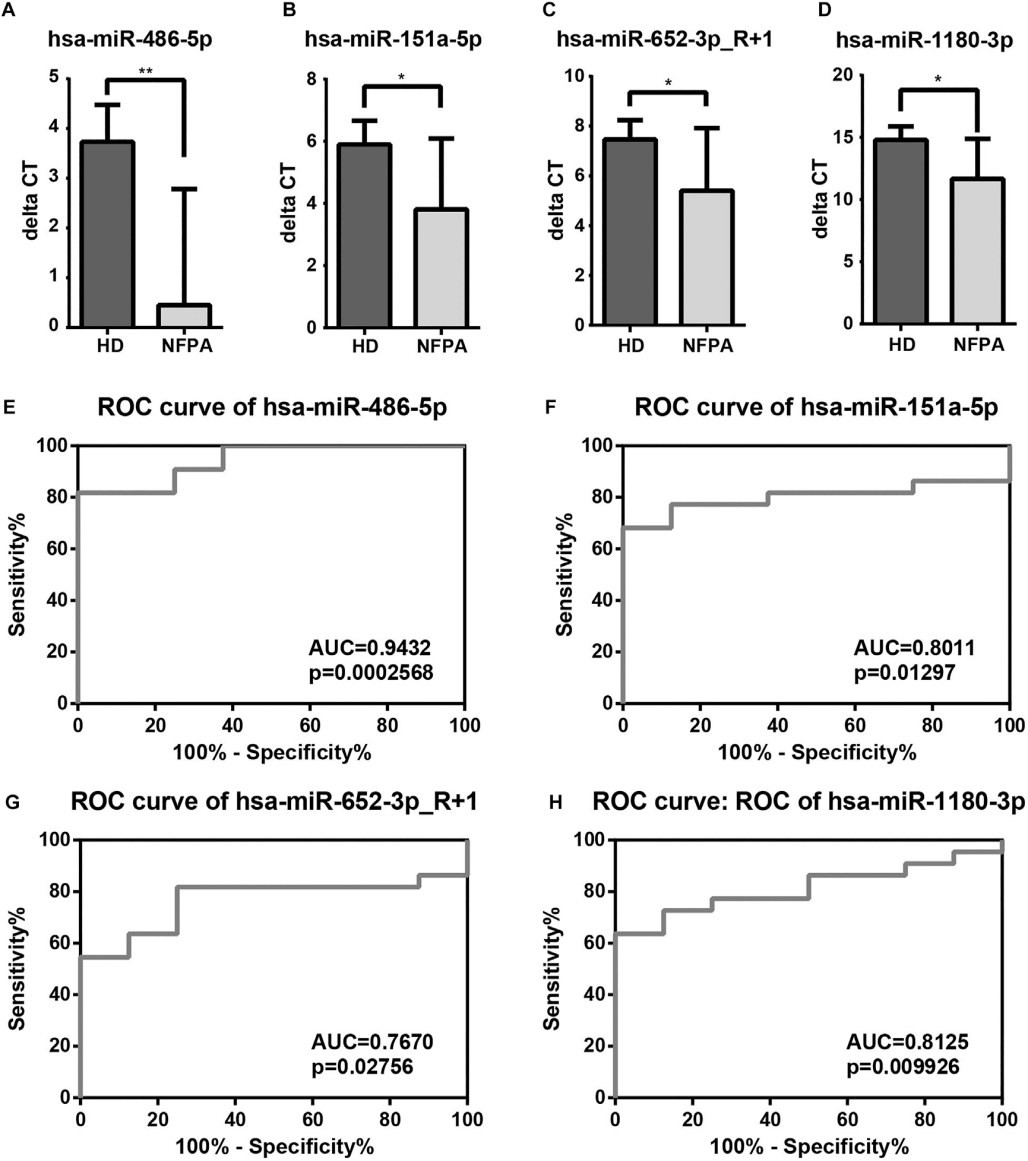
Validation and diagnostic performance of DE-miRNAs. **(A–D)** Among 13 selected DE-miRNAs, hsa-miR-486-5p, hsa-miR-151a-5p, hsa-miR-652-3p_R+1, and hsa-miR-1180-3p were up-regulated in NFPAs. **(E–H)** ROC curves revealed that hsa-miR-486-5p was the most competent biomarker for NFPA screening than hsa-miR-151a-5p, hsa-miR-652-3p_R+1, and hsa-miR-1180-3p.

By miRNA microarray, hsa-miR-486-5p, hsa-miR-151a-5p, hsa-miR-652-3p_R+1, and hsa-miR-1180-3p were promising biomarkers for NFPA. The mean ΔCT value of the HDs group was relatively higher than NFPA patients (3.73 ± 0.75 vs. 0.45 ± 2.34, *p* = 0.0006), which indicated the overexpression of hsa-miR-486-5p in NFPA patients (Figure 4A). hsa-miR-151a-5p, hsa-miR-652-3p_R+1, and hsa-miR-1180-3p were also up-regulated in NFPA patients evidenced by lower ΔCT value compared with HDs (Figures 4B–D). Although hsa-miR-151a-5p presented significant correlation with hsa-miR-486-5p (*r* = 0.7304, *p* < 0.0001), hsa-miR-486-5p (AUC, 0.9432) showed better ability for screening of NFPA than hsa-miR-151a-5p, hsa-miR-652-3p_R+1, and hsa-miR-1180-3p in ROC curves (AUC, 0.8011, 0.7670, and 0.8125, respectively). When enrolled the above-mentioned miRNAs in binary logistic regression analysis, hsa-miR-486-5p was the only independent factor for NFPA diagnosis (OR = 0.125, *p* = 0.027). All together, these results indicated that hsa-miR-486-5p was the most suitable biomarker for perioperative management of NFPA.

### Prospective Follow-Up of Non-Functional Pituitary Adenoma Patients in Validation Cohort

**FIGURE 5:**
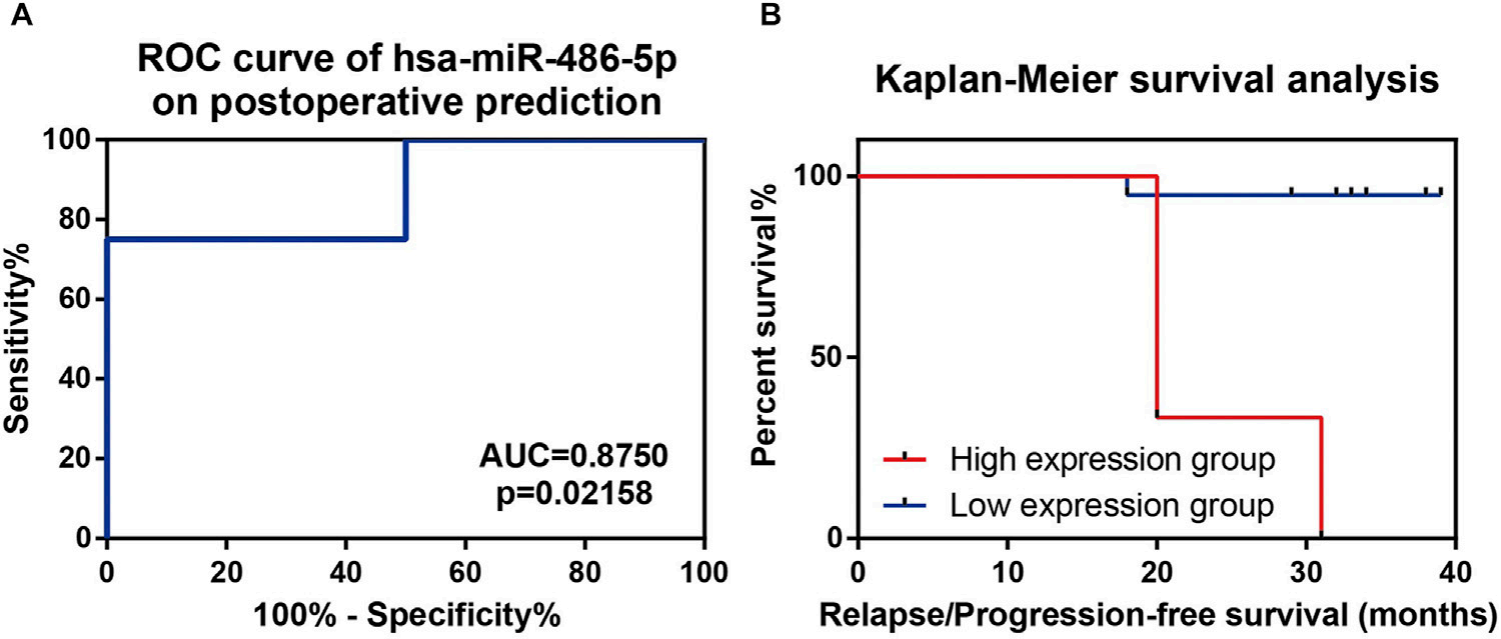
Prospective follow-up of NFPA patients in validation cohort. **(A)** ROC curve indicated that hsa-miR-486-5p had relatively high efficiency in prognosis prediction. **(B)** Kaplan–Meier survival analysis indicated lower ΔCt group (higher hsa-miR-486-5p expression) showed significant lower progression/relapse-free survival rate.

To further investigate the underlying application of exosomal miRNA biomarkers in prognosis prediction, NFPA patients in validation cohort underwent a prospective follow-up for a median of 33 months (range, 18∼39 months). During the follow-up period, 2 out of 16 patients with GTR suffered tumor relapse, and two residuals progressed but one reduced in size for subtotal resected patients (Supplementary Table S3). Compared with relapse/progression-free patients, relapse/progression patients had lower ΔCt values of hsa-miR-486-5p (−2.87 ± 2.72 vs. 1.19 ± 1.50, *p* = 0.0004). However, other validated exosomal miRNAs as well as clinicopathological factors were consistent between two groups (Table 1). The optimal cutoff point of hsa-miR-486-5p expression (ΔCt values) on prognosis prediction was -2.484 by ROC curve (AUC = 0.8750, *p* = 0.02158, Figure 5A). So, NFPA patients were divided into high expression group (ΔCt < −2.484) and low expression group (ΔCt ≥ −2.484). Cox hazard regression analysis indicated that hsa-miR-486-5p expression was the only predictor for NFPA prognosis (HR = 0.542, *p* = 0.014). Further Kaplan–Meier survival analysis revealed that patients with high hsa-miR-486-5p expression suffered worse prognosis (Figure 5B). The 3-year relapse/progression-free survival rate of the low expression group was 94.74% but decreased to 0.0% (*p* < 0.0001) for the high expression group.

**TABLE 1:** Follow-up outcomes of NFPA patients

	Relapse/progression	Relapse/progression-free	*p* value
No. of patients	4	18	
Gender (female, %)	0.0	50.0	0.1150
Age at diagnosis (year)	49.25 ± 7.89	54.72 ± 10.19	0.3281
Knosp grade (1/2/3/4)	0/3/0/1	5/7/4/2	0.3337
Maximal tumor diameter (cm)	3.04 ± 0.51	2.75 ± 0.82	0.5160
Surgical approach (microscopic, %)	100.0	55.6	0.2536
Gross total resection %	50.0	77.8	0.2919
Pathologic subtype			
*Null cell*	2	7	0.8638
*Gonadotroph*	1	6	
*Plurihormonal*	1	3	
*Corticotroph*	0	2	
Relapse/progression-free survival (months)	22.25 ± 5.91	34.00 ± 3.14	**<0.0001**
hsa-miR-1180-3p expression (ΔCt)	11.25 ± 3.52	11.76 ± 3.26	0.7859
hsa-miR-486-5p expression (ΔCt)	−2.87 ± 2.72	1.19 ± 1.50	**0.0004**
has-miR652-3p_R+1 (ΔCt)	4.02 ± 3.88	5.72 ± 2.14	0.2311
hsa-miR-151a-5p (ΔCt)	2.42 ± 3.81	4.12 ± 1.82	0.1848

Data were presented as mean ± standard deviation (SD). Positive results were highlighted in bold.

**FIGURE 6:**
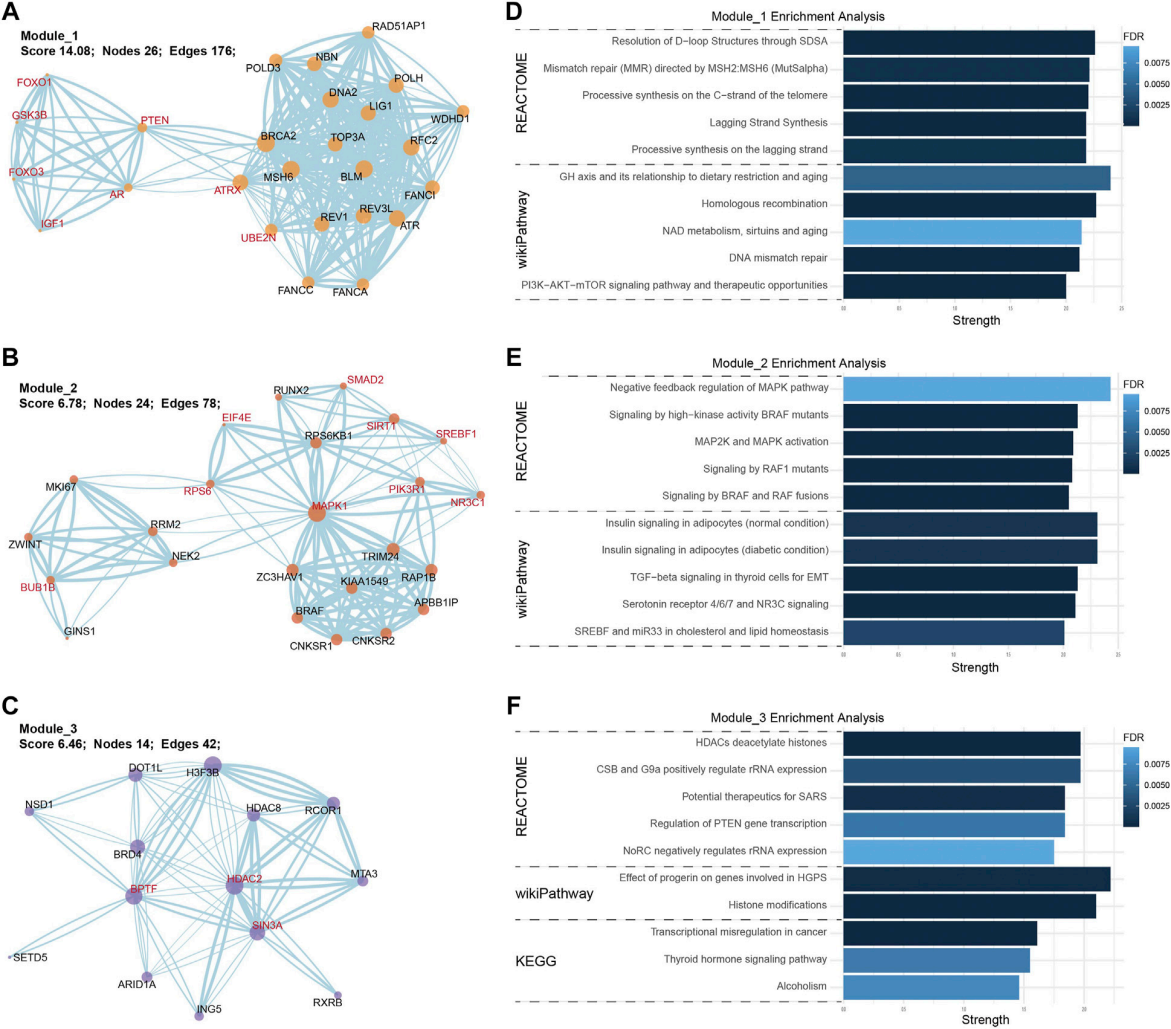
PPI network and enrichment analysis of hsa-miR-486-5p targeted transcripts. The top 3 core PPI modules were built by MCODE plugin. **(A)** Module_1 consisted of 26 nodes and 176 edges; **(B)** Module_2 consisted of 24 nodes and 78 edges; **(C)** Module_3 consisted of 14 nodes and 42 edges (hub genes were highlighted in red). In enrichment analysis, module_1 was enriched in DNA structure-related terms **(D)**, module_2 was enriched in MAPK signaling pathways **(E),** and module_3 was enriched in epigenetic terms **(F)**.

Except for the predictive value of hsa-miR-486-5p, we also constructed a PPI network to illustrate the potential biological function of exosomal hsa-miR-486-5p in NFPA patients. A total of 1235 transcripts were identified as the target of hsa-miR-486-5p. PPI network was analyzed by Cytoscape, and the top 50 nodes with high degree, high betweenness, and closeness were selected by cytoHubba plugin. Thirty overlapped nodes in Venn diagram were identified as significant hub genes (Supplementary Figure S1, Supplementary Table S4). Moreover, MCODE plugin was used to select the most important modules. The top 3 significant modules consisted of 26, 24, and 14 nodes, respectively (Figures 6A–C). Module_1 with 8 hub genes was mainly enriched in DNA structure-related terms, like resolution of D-loop structures through synthesis-dependent strand annealing (SDSA), homologous recombination, and DNA mismatch repair (Figure 6D). Module_2 with 9 hub genes was mainly enriched in signal transduction pathways, like MAPK pathway and insulin signaling (Figure 6E). Module_3 with 3 hub genes was mainly enriched in epigenetic terms, like HDACs deacetylate histones, histone modifications, and transcriptional misregulation in cancer (Figure 6F). These results indicated that hsa-miR-486-5p might affect multiple processes by regulating the core PPI network.

## Discussion

PAs account for about 10% of all intracranial tumors, and NFPA is one of the most common subtypes (Ntali and Wass, 2018). Although most PAs are benign lesions, the prognosis is far from satisfactory. Indeed, more than 40% of NFPAs invade the dura, bone, and cavernous sinuses, which are termed invasive adenomas (Lv et al., 2018a). Meanwhile, a significant number of cases are aggressive adenomas characterized by invasiveness, rapid growth, early recurrence after surgery, and resistance to conventional treatment (Di Ieva et al., 2014). Tumor size and invasiveness are two most relevant factors that affect GTR rate and consequent disease-free survival after surgery (Trouillas et al., 2013; Lv et al., 2018b). Therefore, early detection of NFPAs is one of the most efficient methods for prognosis improvement. Patients with FPAs usually register to medical intervention for menstrual disorders, acral overgrowth, hyperthyroidism, or Cushing syndrome. These clinical clues could lead to further screening for endocrine hormones which are crucial for diagnosis of FPAs. So, hormones like GH, TSH, and PRL are competent biomarkers for FPA screening. However, the absence of reliable biomarker for NFPA partially leads to the consequence that patients with NFPAs usually bear invasive macroadenomas when they require medical care (Raverot et al., 2014). Thus, researchers have been making efforts to address this issue. Circulating tumor cells (CTCs) could be detected in PA patients, but CTCs in peripheral blood are not confident biomarkers for benign PAs for reasons that CTCs are present in 11.1% of patients in the tumor interstitial vascular compartment (Hua et al., 2018). Investigation focused on circulating miRNAs demonstrates that miR-1260b can distinguish PA patients from normal control (Németh et al., 2019). Also, the authors detect the miRNA expression in exosomes, but conclude that there is no significant difference between pre- and post-operative samples. In our study, also focused on miRNA expression, we find that exosomal miRNAs were differentially expressed between HDs and NFPA patients. First, 54 DE-miRNAs were identified by NGS. Further validation proved that exosomal hsa-miR-486-5p, hsa-miR-151a-5p, hsa-miR-652-3p_R+1, and hsa-miR-1180-3p were promising biomarkers for NFPAs. Of note, the overexpression of exosomal hsa-miR-486-5p in NFPAs was consistent in NGS and microarray results. ROC curve of hsa-miR-486-5p also indicated that it was a potential biomarker worthy of further investigation (AUC = 0.9432). Besides, another study investigates the protein expression of serum exosomes in NFPA patients, and confirms that higher expressions of folate receptor 1 and epithelial cell adhesion molecule in non-invasive NFPA, which could serve as biomarkers for evaluation of invasiveness (Wang et al., 2019). In our study, sequence cohort is consisted of three invasive and three non-invasive NFPAs. However, NGS results concluded that the exosomal miRNA profile was consistent among invasive and non-invasive NFPAs.

Although most of PAs are benign, risk assessment of postoperative progression of NFPAs is always a crucial issue for postoperative management. Tumor size and invasiveness are well-known risk factors for relapse (Dallapiazza et al., 2015; Lv et al., 2018a). Specific pathological subtypes, termed high-risk adenoma in the most recent World Health Organization (WHO) classification (Lopes, 2017), also tend to relapse in the early stage after surgery. Taken together, clinicopathological grade system was proposed in 2013 (Trouillas et al., 2013), and proved to be a useful approach in consequent study (Lelotte et al., 2018). In our previous work, a modified grade system improves the predictive efficiency for PAs (Lv et al., 2018b). From the aspect of molecular pathology, USP8, TP53 (Uzilov et al., 2021), phosphohistone-H3 (Li et al., 2020), FAM90A1, and ING2 (Cheng et al., 2020) are reported to be prognostic factors for PAs. All these genes or proteins are underlying prognostic factors for PAs at the postoperative stage. In our study, prospective follow-up allows us to inspect the predictive value of exosomal miRNAs in prognosis of NFPA patients. After a median follow-up of 33 months, hsa-miR-486-5p was proved to be competent for prognostic prediction. Patients with high hsa-miR-486-5p expression (ΔCt < −2.484) suffered significant higher risk for tumor relapse or residual progression. Our findings indicate that preoperative assessment of exosomal hsa-miR-486-5p level may facilitate an individualized treatment strategy that NFPAs with high exosomal hsa-miR-486-5p expression may submit to more radical surgical methods or earlier adjuvant therapies after surgery.

From the functional aspect of miRNAs, we uncovered a global down-regulation of exosomal miRNAs in NFPAs, which was consistent with the miRNA profiles in tumor tissues (Bottoni et al., 2007) and plasma samples of PAs (Németh et al., 2019). Actually, miRNAs repress gene expression by degradation of targeted mRNA. Thus, global down-regulation of miRNAs in PA tissue and blood indicated that tumorigenesis of PAs might be associated with miRNAs down-regulation and consequent targeted oncogenes activation. Meanwhile, allowing for the biological role of exosome (Kalluri, 2016), exosomes in peripheral blood are potential regulatory drivers of tumor progression and function of endocrine glands. After all, miRNAs have a regulative role in the hypothalamic-pituitary-gonadal axis (Cao et al., 2018). Regarding hsa-miR-486-5p, it has been investigated in non-small cell lung cancer. hsa-miR-486-5p inhibits tumor growth and improves chemotherapy sensitivity by targeting PIK3R1 (phosphoinositide-3-kinase regulatory subunit 1) and TWF1 (twinfilin actin binding protein 1), respectively (Jin et al., 2019; Tian et al., 2019). Exosomal hsa-miR-486-5p is also a biomarker for high-risk rectal cancer (Bjørnetrø et al., 2019). With respect to PAs, hsa-miR-486-5p is up-regulated in bromocriptine-resistant prolactinomas (Wu et al., 2014). Nevertheless, the function of up-regulated hsa-miR-486-5p in bromocriptine-resistant prolactinomas as well as NFPA has not been analyzed. By bioinformatics methods, we constructed a PPI network of hsa-miR-486-5p targeted transcripts, finding that the core PPI modules mainly affected epigenetic events, DNA structure regulation, and MAPK signaling pathways. Pituitary is a highly vascularized endocrine gland. Thus, these exosomes have the likelihood of regulating tumorigenesis of NFPA in a paracrine manner. Thus, according to our data, exosomal hsa-miR-486-5p might regulate tumor progression by epigenetic regulation of MAPK signaling pathways, which deserved further laboratory investigation.

The current study also suffered from some limitations. First, allowing for the relatively small sample size, the results need further verification. Second, due to the unavailability of normal pituitary tissue, we could not confirm if the DE-miRNAs were originated from NFPA, although comparing NFPA tissue with normal pituitary by miRNA sequencing was initially on our schedule. Last, only preoperative blood samples were analyzed in this study. In our view, it was more convincing if exosomal miRNAs were preoperatively up-/down-regulated but decreased/increased after surgery.

## Conclusion

In conclusion, exosomal miRNA profiling in peripheral blood is a novel and promising approach for perioperative management of NFPAs. Exosomal hsa-miR-486-5p, hsa-miR-151a-5p, hsa-miR-652-3p_R+1, and hsa-miR-1180-3p are candidate biomarkers for diagnosis and screening of NFPAs. More importantly, prospective follow-up reveals that hsa-miR-486-5p can be regarded as a significant predictor for prognosis of NFPAs.

## Data Availability

The raw miRNA-seq data can be found in the National Genomics Data Center (https://ngdc.cncb.ac.cn/) through BioProject database (Accession number: PRJCA006867) or GSA-human database (Accession number: HRA001446). Other raw data supporting the conclusions and R codes used in this article will be made available by correspondent authors on reasonable requests, without undue reservation.

## Supplementary Material

Supplementary Figure S1 Venn diagram of hub genes cytoHubba plugin selected the top 50 nodes with high degree, high closeness, and high betweenness. Interactive Venn diagram identified the overlapped hub genes.
