# Supplementary figures and images for: Exosomal miRNA Profiling is a Potential Screening Route for Non-Functional Pituitary Adenoma
Source: Front Cell Dev Biol. 2022 Jan 18;9:771354. doi: 10.3389/fcell.2021.771354 (PMC8804500; doi:10.3389/fcell.2021.771354)

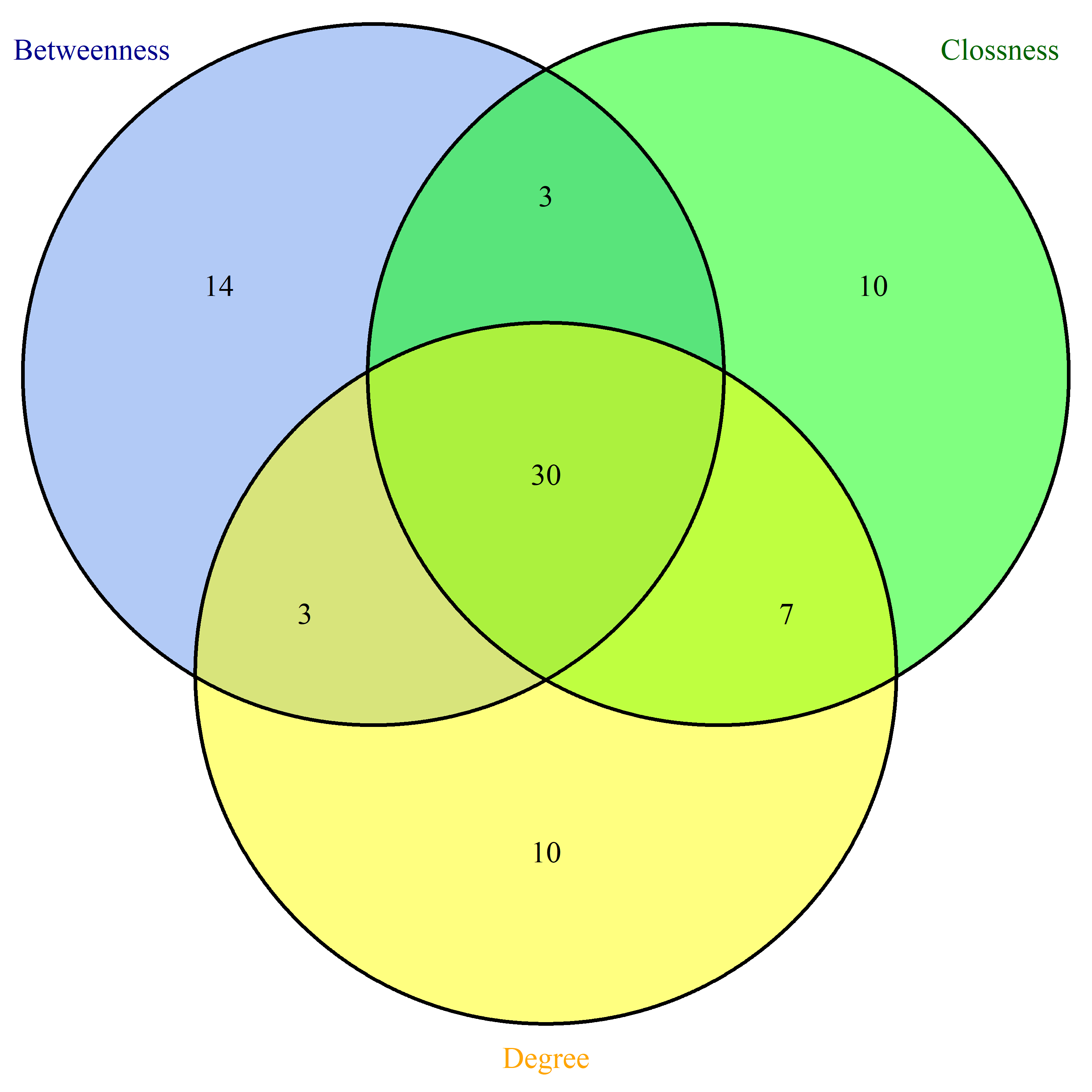

Supplement: Supplementary file 1 [file Image1.TIFF]
