# Supplementary material for: Exosomal miRNA Profiling is a Potential Screening Route for Non-Functional Pituitary Adenoma
Source: Front Cell Dev Biol. 2022 Jan 18;9:771354. doi: 10.3389/fcell.2021.771354 (PMC8804500; doi:10.3389/fcell.2021.771354)
Supplement: Supplementary file 2 [file Table1.DOCX]

| Supplementary Table.1 Differentially expressed exosomal miRNAs | | | | | | |
| --- | --- | --- | --- | --- | --- | --- |
| miR name | miR sequence | Phenotype | Regulation | Log2(FC) | P value | Expression level |
| hsa-miR-1180-3p | TTTCCGGCTCGCGTGGGTGTGT | Known | Up | 2.84 | 1.79E-02 | Middle |
| hsa-miR-194-5p | TGTAACAGCAACTCCATGTGGA | Known | Up | 1.87 | 2.42E-02 | Middle |
| hsa-miR-199b-5p_R-1 | CCCAGTGTTTAGACTATCTGTT | Variation | Up | 1.05 | 3.79E-02 | Middle |
| hsa-miR-486-5p | TCCTGTACTGAGCTGCCCCGAG | Known | Up | 0.80 | 3.78E-02 | high |
| hsa-miR-132-5p | ACCGTGGCTTTCGATTGTTACT | Known | Down | -3.14 | 3.54E-04 | Middle |
| hsa-miR-758-3p_1ss22CT | TTTGTGACCTGGTCCACTAACT | Variation | Down | -2.33 | 3.21E-02 | Middle |
| hsa-miR-136-5p_R-1 | ACTCCATTTGTTTTGATGATGG | Variation | Down | -2.24 | 1.84E-03 | Middle |
| hsa-miR-181c-3p_L-1R+1 | ACCATCGACCGTTGAGTGGACC | Variation | Down | -2.17 | 1.59E-04 | Middle |
| hsa-miR-1908-5p | CGGCGGGGACGGCGATTGGTC | Known | Down | -2.16 | 1.45E-03 | Middle |
| hsa-miR-628-5p | ATGCTGACATATTTACTAGAGG | Known | Down | -2.13 | 2.68E-03 | Middle |
| hsa-miR-493-3p | TGAAGGTCTACTGTGTGCCAGG | Known | Down | -2.00 | 4.53E-02 | Middle |
| hsa-miR-376c-3p | AACATAGAGGAAATTCCACGT | Known | Down | -2.00 | 5.57E-03 | Middle |
| hsa-miR-1304-3p_1ss13CA | TCTCACTGTAGCATCGAACCCC | Variation | Down | -1.69 | 1.78E-02 | Middle |
| hsa-miR-409-3p | GAATGTTGCTCGGTGAACCCCT | Known | Down | -1.65 | 9.60E-03 | Middle |
| hsa-miR-889-3p | TTAATATCGGACAACCATTGT | Known | Down | -1.63 | 2.15E-02 | Middle |
| hsa-miR-181a-2-3p | ACCACTGACCGTTGACTGTACC | Known | Down | -1.61 | 2.86E-02 | Middle |
| hsa-miR-370-3p | GCCTGCTGGGGTGGAACCTGGT | Known | Down | -1.57 | 1.38E-02 | Middle |
| hsa-miR-539-3p | ATCATACAAGGACAATTTCTTT | Known | Down | -1.50 | 2.34E-02 | Middle |
| hsa-miR-11400_1ss20TC | TCGGCTGTGTATCTCTGTGCC | Variation | Down | -1.49 | 5.15E-03 | Middle |
| hsa-miR-7977_1ss6AG | TTCCCGGCCAACGCACCA | Variation | Down | -1.46 | 1.75E-02 | Middle |
| hsa-miR-4433b-5p_R+1 | ATGTCCCACCCCCACTCCTGTT | Variation | Down | -1.39 | 2.59E-02 | Middle |
| hsa-miR-493-5p | TTGTACATGGTAGGCTTTCATT | Known | Down | -1.34 | 3.81E-04 | Middle |
| hsa-miR-2355-5p_R+1 | ATCCCCAGATACAATGGACAAT | Variation | Down | -1.28 | 3.11E-02 | Middle |
| hsa-miR-628-3p | TCTAGTAAGAGTGGCAGTCGA | Known | Down | -1.25 | 5.28E-03 | Middle |
| hsa-miR-146a-5p | TGAGAACTGAATTCCATGGGTT | Known | Down | -1.18 | 1.12E-02 | High |
| hsa-miR-98-5p | TGAGGTAGTAAGTTGTATTGTT | Known | Down | -1.15 | 6.37E-04 | High |
| hsa-miR-151a-3p | CTAGACTGAAGCTCCTTGAGG | Known | Down | -1.07 | 1.07E-02 | High |
| hsa-miR-425-3p_L+1R-1 | CATCGGGAATGTCGTGTCCGCC | Variation | Down | -0.99 | 4.87E-03 | Middle |
| hsa-miR-26a-5p | TTCAAGTAATCCAGGATAGGCT | Known | Down | -0.96 | 5.08E-03 | High |
| hsa-let-7f-5p | TGAGGTAGTAGATTGTATAGTT | Known | Down | -0.92 | 1.04E-02 | High |
| hsa-miR-151a-5p | TCGAGGAGCTCACAGTCTAGT | Known | Down | -0.91 | 1.64E-02 | High |
| hsa-miR-652-3p_R+1 | AATGGCGCCACTAGGGTTGTGA | Variation | Down | -0.89 | 9.23E-03 | High |
| hsa-miR-27a-3p | TTCACAGTGGCTAAGTTCCGC | Known | Down | -0.87 | 1.59E-03 | High |
| hsa-miR-30b-5p | TGTAAACATCCTACACTCAGCT | Known | Down | -0.81 | 6.36E-03 | High |
| hsa-miR-191-5p_R-1 | CAACGGAATCCCAAAAGCAGCT | Variation | Down | -0.74 | 1.50E-02 | High |
| hsa-miR-501-3p | AATGCACCCGGGCAAGGATTCT | Known | Down | -0.73 | 4.66E-02 | Middle |
| hsa-miR-30e-3p_1ss22CT | CTTTCAGTCGGATGTTTACAGT | Variation | Down | -0.71 | 4.65E-02 | Middle |
| hsa-let-7i-5p | TGAGGTAGTAGTTTGTGCTGTT | Known | Down | -0.69 | 2.12E-02 | High |
| hsa-miR-30e-5p_R+2 | TGTAAACATCCTTGACTGGAAGCT | Variation | Down | -0.61 | 3.98E-02 | High |
| hsa-miR-30d-5p_R+2 | TGTAAACATCCCCGACTGGAAGCT | Variation | Down | -0.56 | 2.11E-02 | High |
| PC-5p-60318_72 | ATCCTGGGGCTGGAGTAGG | Predicted | Up | 4.72 | 4.24E-02 | Middle |
| PC-5p-29646_172 | TCTTGAGTATGGAAGAGGT | Predicted | Up | 4.10 | 3.49E-02 | Middle |
| PC-3p-35108_142 | TGACTTGTGGCTAGGGAA | Predicted | Up | 3.22 | 2.74E-02 | Middle |
| PC-3p-20319_264 | GCAGGTCCCAAGGGTATG | Predicted | Up | 3.09 | 3.51E-02 | Middle |
| PC-3p-24305_216 | CAGTAGCTCGAGGAAAAG | Predicted | Up | 2.71 | 4.00E-02 | Middle |
| PC-5p-2148_3376 | AGCGGTACGTGAGTTGGG | Predicted | Up | 2.62 | 3.51E-02 | High |
| PC-3p-15275_361 | CTTGTGGAGCCATCCTTGA | Predicted | Up | 2.49 | 1.31E-02 | Middle |
| PC-5p-47133_98 | TTTACTAAAAACACAGGTC | Predicted | Up | 2.47 | 2.44E-02 | Middle |
| PC-3p-27654_187 | GTGGTAGGCTTTGAAGCA | Predicted | Up | 2.24 | 4.96E-02 | Middle |
| PC-3p-11519_487 | TCTTCGTGGATGTCTAACC | Predicted | Up | 2.20 | 2.52E-02 | Middle |
| PC-3p-79529_49 | ATGCTGACATGAGTAACG | Predicted | Up | 2.18 | 4.29E-02 | Middle |
| PC-5p-22617_235 | GGGTGTTGGATGTCCCTGTTGCT | Predicted | Up | 1.84 | 2.38E-02 | Middle |
| PC-5p-19247_281 | AATCCTCTCGTGGACACC | Predicted | Up | 1.51 | 3.73E-02 | Middle |
| PC-5p-20655_259 | GCAGGCATGAGTAGCGAAA | Predicted | Up | 1.29 | 3.72E-02 | Middle |
| miR: miRNA; FC: fold change | | | | | | |
