# Supplementary material for: Exosomal miRNA Profiling is a Potential Screening Route for Non-Functional Pituitary Adenoma
Source: Front Cell Dev Biol. 2022 Jan 18;9:771354. doi: 10.3389/fcell.2021.771354 (PMC8804500; doi:10.3389/fcell.2021.771354)
Supplement: Supplementary file 3 [file Table2.DOCX]

| Supplementary Table.2 Clinical and pathological characteristics of NFPAs | | | | | | | |
| --- | --- | --- | --- | --- | --- | --- | --- |
| Patients | Gender/Age | Approach | AP diameter/ Height/ Width (cm) | Pathological Subtype | Ki-67  (%) | Knosp Grade | Endocrine Disorders |
| Sequence cohort |  |  |  |  |  |  |  |
| 1 | Male/68y | Microscopic | 1.82/2.21/2.04 | Gonadotroph | 1.6 | 2 |  |
| 2 | Female/46y | Microscopic | 3.10/3.14/2.86 | Plurihormonal | 5.0 | 2 |  |
| 3 | Male/62y | Microscopic | 2.16/2.10/1.58 | Gonadotroph | 1.0 | 1 |  |
| 4 | Male/43y | Microscopic | 2.22/2.46/3.13 | Null cell | 7.6 | 4 |  |
| 5 | Male/61y | Microscopic | 2.86/3.26/2.82 | Gonadotroph | 0.6 | 3 |  |
| 6 | Female/46y | Microscopic | 1.87/1.62/2.35 | Plurihormonal | 2.0 | 3 |  |
| Validation cohort |  |  |  |  |  |  |  |
| 1 | Male/51y | Microscopic | 1.56/1.86/2.31 | Plurihormonal | 2.0 | 2 | HPA |
| 2 | Male/71y | Endoscopic | 2.39/2.01/2.07 | Plurihormonal | 5.0 | 2 | None |
| 3 | Male/36y | Endoscopic | 2.07/2.00/2.70 | Null cell | 1.0 | 1 | HPG |
| 4 | Male/41y | Microscopic | 2.31/3.76/3.64 | Null cell | 3.0 | 2 | HPA HPT |
| 5 | Male/56y | Microscopic | 1.25/1.43/1.31 | Null cell | 1.5 | 1 | HPA HPT |
| 6 | Male/56y | Microscopic | 1.95/2.80/2.31 | Null cell | 2.0 | 2 | HPA HPT |
| 7 | Male/52y | Endoscopic | 3.94/4.05/2.29 | Null cell | 2.0 | 2 | None |
| 8 | Male/44y | Microscopic | 1.88/2.58/2.97 | Null cell | 1.5 | 4 | HyperPRL HPA |
| 9 | Male/56y | Microscopic | 1.51/2.61/1.89 | Gonadotroph | 3 | 2 | HPA |
| 10 | Male/55y | Microscopic | 1.68/2.13/2.30 | Gonadotroph | 3 | 2 | HyperPRL HPA HPT |
| 11 | Male/59y | Endoscopic | 2.31/3.82/2.97 | Gonadotroph | 0.5 | 2 | HPA |
| 12 | Male/72y | Microscopic | 1.92/3.67/2.22 | Gonadotroph | 0.8 | 3 | HPA |
| 13 | Male/53y | Endoscopic | 1.82/2.59/1.74 | Gonadotroph | 1.2 | 3 | HPA HPG |
| 14 | Female/30y | Microscopic | 2.03/2.40/2.44 | Plurihormonal | 0.8 | 2 | HyperPRL HPA |
| 15 | Female/48y | Microscopic | 1.72/2.28/2.68 | Null cell | 4.0 | 3 | HyperPRL |
| 16 | Female/54y | Endoscopic | 3.10/4.05/3.80 | Null cell | 1.5 | 4 | HyperPRL HPA HPT HPG |
| 17 | Female/61y | Microscopic | 1.17/1.48/1.47 | Null cell | 1.5 | 1 | None |
| 18 | Female/55y | Endoscopic | 2.16/1.92/3.15 | Null cell | 1.5 | 3 | GH HPA |
| 19 | Female/64y | Microscopic | 2.36/2.60/2.10 | Gonadotroph | 2 | 1 | HyperPRL HPT HPG |
| 20 | Female/56y | Microscopic | 2.00/2.10/3.00 | Gonadotroph | 3.0 | 2 | HyperPRL HPA HPT HPG |
| 21 | Female/53y | Microscopic | 1.95/3.30/2.10 | Corticotroph | 0.7 | 4 | HPA HPG |
| 22 | Female/59y | Endoscopic | 1.52/1.23/1.54 | Corticotroph | 1.0 | 1 | HPA |
| AP: anteroposterior; HPA: hypothalamus pituitary adrenal axis; HPG: hypothalamus pituitary gonadal axis; HPT: hypothalamus pituitary thyroid axis; HyperPRL: hyperprolactinemia; GH: growth hormone deficiency | | | | | | | |
