# Supplementary material for: Exosomal miRNA Profiling is a Potential Screening Route for Non-Functional Pituitary Adenoma
Source: Front Cell Dev Biol. 2022 Jan 18;9:771354. doi: 10.3389/fcell.2021.771354 (PMC8804500; doi:10.3389/fcell.2021.771354)
Supplement: Supplementary file 5 [file Table4.docx]

| Supplementary Table.4 Hub genes of hsa-miR-486-5p | | | | |
| --- | --- | --- | --- | --- |
| No. | Official Gene Symbol | Betweenness | Closeness | Degree |
| 1 | MAPK1 | 79338.50416 | 462.8333333 | 64 |
| 2 | PTEN | 75436.03047 | 478.2333333 | 74 |
| 3 | AR | 53072.38569 | 459.4 | 60 |
| 4 | SIRT1 | 47888.55152 | 455.95 | 59 |
| 5 | UBXN7 | 44606.21625 | 402.4166667 | 35 |
| 6 | PIK3R1 | 40034.40119 | 439.0333333 | 48 |
| 7 | BPTF | 36332.6109 | 425.2833333 | 51 |
| 8 | HDAC2 | 31596.77636 | 440.5166667 | 57 |
| 9 | GSK3B | 27456.68272 | 429.4166667 | 37 |
| 10 | GOLPH3 | 26881.23909 | 386.0333333 | 29 |
| 11 | EIF4E | 25857.52107 | 421.6333333 | 40 |
| 12 | RPS6 | 25191.70439 | 422.4833333 | 38 |
| 13 | SMAD2 | 25186.67114 | 436.3666667 | 45 |
| 14 | SHH | 24368.72871 | 414.3166667 | 41 |
| 15 | ATRX | 23977.6042 | 428.3166667 | 44 |
| 16 | SREBF1 | 23804.9682 | 415.7166667 | 33 |
| 17 | UBE2N | 22316.34639 | 394.75 | 36 |
| 18 | FMR1 | 20090.70117 | 405.1333333 | 29 |
| 19 | NR3C1 | 19425.5763 | 422.55 | 35 |
| 20 | TERT | 17752.27761 | 420.2833333 | 35 |
| 21 | FOXO3 | 17708.67858 | 437.9166667 | 44 |
| 22 | IGF1 | 15826.80852 | 430.6833333 | 38 |
| 23 | BUB1B | 15304.93413 | 392.7 | 41 |
| 24 | ICAM1 | 14861.69214 | 386.1166667 | 29 |
| 25 | USP7 | 14697.4369 | 405.7666667 | 27 |
| 26 | SRSF1 | 14568.57224 | 401.6333333 | 35 |
| 27 | DNMT3A | 13259.17406 | 401.2166667 | 31 |
| 28 | IKZF1 | 12934.06246 | 396.9333333 | 29 |
| 29 | SIN3A | 12793.49919 | 414.1833333 | 37 |
| 30 | FOXO1 | 11854.38516 | 434.35 | 38 |
| Data were generated by cytoHubba Plugin of Cytoscope software. | | | | |
